# A comprehensive multiomics approach reveals that high levels of sphingolipids in cardiac cachexia adipose tissue are associated with inflammatory and fibrotic changes

**DOI:** 10.1186/s12944-023-01967-0

**Published:** 2023-12-01

**Authors:** Yiwei Qu, Yong Wang, Tao Wu, Xue Liu, Huaizhe Wang, Dufang Ma

**Affiliations:** 1 Shandong University of Traditional Chinese Medicine, Jinan, China; 2 Affiliated Hospital of Shandong University of Traditional Chinese Medicine, Jinan, China

**Keywords:** Cardiac cachexia, Adipose tissue remodelling, Transcriptomics, Metabolomics, Lipidomics, Sphingolipids, Inflammation, Macrophages, Fat fibrosis, Adipose tissue dysfunction

## Abstract

Cardiac cachexia is a deadly consequence of advanced heart failure that is characterised by the dysregulation of adipose tissue homeostasis. Once cachexia occurs with heart failure, it prevents the normal treatment of heart failure and increases the risk of death. Targeting adipose tissue is an important approach to treating cardiac cachexia, but the pathogenic mechanisms are still unknown, and there are no effective therapies available. Transcriptomics, metabolomics, and lipidomics were used to examine the underlying mechanisms of cardiac cachexia. Transcriptomics investigation of cardiac cachexia adipose tissue revealed that genes involved in fibrosis and monocyte/macrophage migration were increased and strongly interacted. The ECM-receptor interaction pathway was primarily enriched, as shown by KEGG enrichment analysis. In addition, gene set enrichment analysis revealed that monocyte chemotaxis/macrophage migration and fibrosis gene sets were upregulated in cardiac cachexia. Metabolomics enrichment analysis demonstrated that the sphingolipid signalling pathway is important for adipose tissue remodelling in cardiac cachexia. Lipidomics analysis showed that the adipose tissue of rats with cardiac cachexia had higher levels of sphingolipids, including Cer and S1P. Moreover, combined multiomics analysis suggested that the sphingolipid metabolic pathway was associated with inflammatory-fibrotic changes in adipose tissue. Finally, the key indicators were validated by experiments. In conclusion, this study described a mechanism by which the sphingolipid signalling pathway was involved in adipose tissue remodelling by inducing inflammation and fat fibrosis in cardiac cachexia.

**Supplementary Information:**

The online version contains supplementary material available at 10.1186/s12944-023-01967-0.

## Introduction

Heart failure (HF) is the clinical manifestation of cardiovascular diseases in their terminal stages. Cachexia is the most severe complication of end-stage HF and a risk factor for mortality in patients at this stage of the disease [1]. Cachexia is defined as a 12-month oedema-free weight loss of more than 5%, which is accompanied by the following signs: muscle weakness, fat loss, anorexia, malaise, elevated levels of proinflammatory factors, anaemia, and hypoproteinaemia [2, 3]. Regardless of other criteria, researchers have used a weight loss threshold exceeding 5% as a defining criterion [4, 5]. According to epidemiological research, depending on the definition of cachexia and the study population, the prevalence of cardiac cachexia ranges from 8 to 42% [6]. The occurrence of cachexia increases mortality in patients with chronic HF by 20–40% in 1 year [7–9]. It might be challenging for patients to survive when they lose up to 66% of their optimal body weight [10]. Nevertheless, until now, the underlying pathophysiological mechanisms have not been well elucidated [11].

Adipose tissue is a highly dynamic organ and a significant energy reserve in the body [12]. Excessive fat consumption has been shown to be substantially linked to the severity of cardiac cachexia [13], and reduced fat mass is more strongly associated with cachexia and prognosis than muscle and nonfat mass [14]. Thus, targeting adipose tissue is an important strategy to treat cardiac cachexia.

In adipose tissue, extracellular matrix (ECM) proteins play an essential role by providing structural integrity to adipocytes and serving as mediators of many signalling processes [15]. In obese individuals, it has been revealed that an increase in ECM proteins results in fat fibrosis, which is adipose tissue remodelling [16]. These proteins damage the microenvironment of adipose tissue and decrease its flexibility, which leads to adipose tissue dysfunction and systemic metabolic diseases [17]. Similarly, smaller lipid droplets and more severe fat fibrosis in white adipose tissue were observed in mice with tumour-induced cachexia [18]. Additionally, a clinical study reported that adipose fibrosis was observed in patients with cancer-induced cachexia. These patients exhibited increased expression of collagens and fibronectin compared with cancer patients with stable body weight [19]. These studies indicated that adipose tissue remodelling characterised by fat fibrosis occurred in the context of cachexia, which is related to adipose tissue dysfunction caused by cachexia.

It is well known that inflammation drives fibrosis. A typical clinical trait of cachexia is persistent inflammation, which is similar to adipose tissue in obese conditions. Several proinflammatory factors, such as transforming growth factor-β (TGF-β), interleukin-1β (IL-1β) and interleukin-6 (IL-6), are upregulated in adipose tissue during cachexia, which promotes ECM production [19]. For example, TGF-β-treated adipocytes exhibit increased expression of ECM remodelling proteins [20]. Additionally, immunocyte infiltration, especially macrophage infiltration, was observed in adipose tissue in cancer-induced cachexia [18]. Macrophages can induce fibrosis by recruiting and activating fibroblasts, which differentiate into myofibroblasts and synthesize extracellular matrix [21]. Therefore, chronic inflammation interacts with fat fibrosis to contribute to adipose tissue remodelling in cachexia. However, questions remain regarding the exact molecular mechanisms involved in cachexia-induced adipose tissue remodelling.

**Fig. 1:**
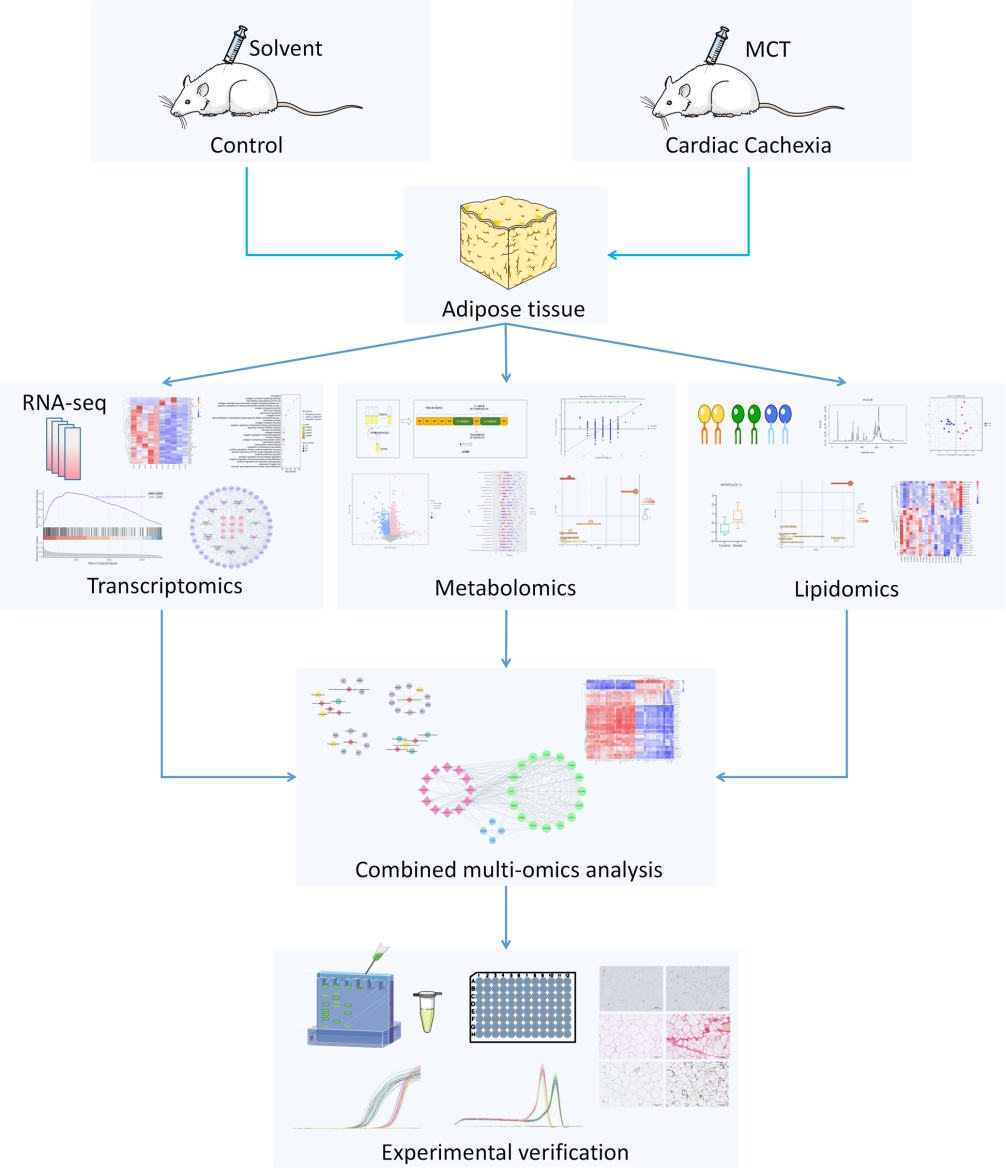
Flowchart of the study

In recent years, multiomics analysis techniques have been increasingly used to clarify the pathogenesis and mechanisms of adipose tissue in metabolic disorders [22, 23]. Through comprehensive use of metabolomics and transcriptomics, David B et al. [24] found that visceral adipose tissue from patients with colorectal cancer exhibited stronger inflammatory signals and higher levels of metabolites with proinflammatory effects than subcutaneous adipose tissue. However, studies of cardiac cachexia using this approach have not been reported. For the first time, transcriptomics, metabolomics, and lipidomics were used to investigate the underlying mechanisms of adipose tissue dysfunction in cardiac cachexia, identify differentially expressed genes (DEGs), metabolites (DEMs), and lipids (DELs), and conduct a correlation network analysis of DEGs and DELs. This study suggests that high levels of sphingolipids impact adipose tissue remodelling in cardiac cachexia primarily by activating inflammation and the onset of fat fibrosis. Figure 1 shows a complete flowchart of this study.

## Materials and methods

### Animals

In this study, pulmonary hypertension caused by monocrotaline (MCT) rapidly developed into HF and cachexia in rats [25]. Compared to that in other animal models, the onset of cachexia in the MCT model is shorter, faster, and more variable, resulting in a more sensitive assay [26]. The Animal Ethics Committee of the Affiliated Hospital of Shandong University of Traditional Chinese Medicine (No. 2021–30) authorised the experimental protocols.

Twenty 6-week-old (180–200 g) SPF-grade Wistar male rats were purchased from Beijing Vital River Laboratory Animal Technology Co., Ltd. (Beijing, China) (Permit No. SCXK(JING) 2021–0011). Throughout the experiment, the rats were fed freely and maintained at 23 °C with constant humidity and a typical circadian rhythm environment. The rats were divided equally into two groups (*n* = 10) one a week of acclimatisation. The control group received an intraperitoneal injection of 3 ml/kg solvent (saline: anhydrous ethanol = 8:2), while the model group was intraperitoneally injected with 60 mg/kg MCT [27]. After MCT injection, the food intake of the control group was the same as that of the model group. Rat body weights were recorded weekly. After 6 weeks, ultrasound cardiography was performed to evaluate the functionality of the left and right ventricles (LV and RV). Relevant indices included left ventricular ejection fraction (LVEF) and left ventricular fractional shortening (LVFS), as well as right ventricular end-diastolic area (RVEDA) and right ventricular fractional area change (RVFAC). RVFAC was calculated as follows: RVFAC = [EDA-ESA (end-systolic area)]/EDA × 100%. A considerable decline in cardiac function and a decrease in body weight to 8.5% of peak body weight were indicators of successful modelling of cardiac cachexia in rats [26]. The tissue was removed after sodium pentobarbital (40 mg/kg, i.p.) was used to anaesthetise each rat. As quickly as possible, the epididymal and inguinal adipose tissue were removed and placed on ice. The total weight of the adipose tissue was measured, and then the epididymal adipose tissue was separated into two equal pieces, one of which was kept in 10% paraformaldehyde for histological analysis, while the other was cryopreserved for multiomics testing and experimental validation.

### mRNA sequencing

An RNeasy mini kit (Qiagen, Germany) was used to extract total RNA, and RNA concentration and quality were evaluated using a Qubit® 3.0 fluorometer (Life Technologies, USA) and a Nanodrop One spectrophotometer (Thermo Fisher Scientific Inc., USA). After analysis on an Agilent 2100 Bioanalyzer (Agilent Technologies Inc., USA), samples with total RNA integrity values greater than 7.0 were selected for sequencing. According to the instructions, a paired-end library was created using the Stranded mRNA-seq Lib Prep Kit for Illumina (ABclonal, China). The mRNA was then purified and disintegrated with divalent cations at 94 °C for 10 min. The RNA fragments were then reverse transcribed into first-strand cDNA. Then, second-strand cDNA was generated using ribonuclease and DNA polymerase. Following the insertion of one 'A' base, these cDNA fragments underwent end repair, and the adapters were subsequently ligated. The cDNA library was prepared by purifying and enriching the PCR products. The library was then quantified using a Qubit® 3.0 Fluorometer (Life Technologies, USA) and validated using an Agilent 2100 Bioanalyzer (Agilent Technologies, USA) to verify the size of the insert and determine the molar concentration. After the library was diluted to 10 pM, the cluster was created with cBot and sequenced on an Illumina NovaSeq 6000 (Illumina, USA).

### LC‒MS metabolomics assay

In this study, nontargeted metabolomics analysis of cardiac cachexia adipose tissue was carried out using liquid chromatography‒mass spectrometry (LC‒MS) (Waters, UPLC; Thermo, Q Exactive) [28, 29]. Then, a volume of 800 μL of methanol (80%) was used to homogenise the samples for 90 s at 65 Hz before the samples were vortexed and agitated to properly blend them. 30 min of sonication was performed at 4 °C, and the samples were then centrifuged at -40 °C for 1 h and vortexed for 30 s. The supernatant was centrifuged at 12,000 rpm for 15 min at 4 °C and then allowed to stand at -40 °C for 1 h. The internal standard was then injected into the supernatant, fully mixed, and subsequently transferred to the injection vial. Detection was performed using an ACQUITY UPLC HSS T3 column (2.1 × 100 mm 1.8 m) on an LC‒MS instrumental analysis platform at 40 °C with a flow rate of 0.3 ml/min. The mobile phases were A (water + 0.05% formic acid) and B (acetonitrile). The elution gradients were as follows: 0–12 min, 95% A, 5% B; 12–13.6 min, 5% A, 95% B; and 13.6–16 min, 95% A, 5% B. The following mass spectrometry detection parameters were used: capillary temperature, 350 °C; electrospray rate, 1 arb; electrospray voltage, 3.0 kV; heater temperature, 300 °C; sheath gas flow rate, 45 arb; auxiliary gas flow rate, 15 arb; tail gas flow rate, 1 arb; and S-Lens RF level, 30%. Full scan (*m/z* range 701,050) with data-dependent secondary mass spectrometry (dd-MS2) (TopN = 10) was used as the scanning mode. Primary mass spectrometry had a resolution of 70,000, and secondary mass spectrometry had a resolution of 17,500. The collision mode used in this study was high-energy collisional dissociation.

### LC‒MS lipidomics assay

The adipose tissue (50 mg) and reagent (chloroform/methanol and fresh water) mixture was vortexed for 1 min at 60 Hz, homogenised for 180 s, and then sonicated for 30 min at 4 °C. The organic phase was extracted and transferred to a fresh test tube after centrifugation and then dried with nitrogen. The supernatant was collected from the injection vial and examined after the organic phase was centrifuged. The chromatographic column was an ACQUITY UPLC BEH C18 (2.1 × 100 mm 1.7 m). The instrumental analysis platform and chromatographic column conditions were the same as those for metabolomics. The elution gradients were set as 0–10.5 min, 70% A, 30% B; 10.5–12.51 min, 100% B; and 12.51–16 min, 70% A, 30% B. The following mass spectrometric detection parameters were used: capillary temperature, 350 °C; electrospray voltage, 3.2 kV; heater temperature, 300 °C; sheath gas flow rate, 45 arb; auxiliary gas flow rate, 15 arb; tail gas flow rate, 1 arb; and S-Lens RF level, 60%. Full scan (*m/z* range 200–1200) with dd-MS2 (TopN = 10) was used as the scanning mode.

### Data analysis

#### Transcriptomics data analysis

FastQC software was used to evaluate the accuracy of each fastq file of sequencing data after the raw sequencing data had been stored as fastq format files, and then the fastq files were aligned to the reference genome using Hisat2 (v. 2.0.5) [30]. SAMtools (v. 1.3.1) was used to sort and convert the sequencing alignment/map (SAM) data into binary alignment/map (BAM) files. The abundance was calculated in terms of fragments per kilobase of exon per million reads mapped (FPKM) [31]. All of the gene segments were counted using the Stringtie program [32], and normalisation was performed using the TMM technique [33]. Differential mRNA expression was analysed using R. For subsequent analysis, DEGs with |log2(FC)|> 1, *Q* value < 0.05, and the mean FPKM of one group > 1 were determined to have significantly altered expression. This criterion was applied to boost the study's sensitivity so that a thorough screening could be performed and potential candidate genes could be chosen for validation using real-time PCR analysis of a larger sample set. Enrichment analysis of DEGs was performed by Gene Ontology (GO) and Kyoto Encyclopaedia of Genes and Genomes (KEGG) pathway analysis. Several significant DEGs were incorporated into the STRING database to generate the interaction relationship data. After importing the data into Cytoscape (v. 3.9.0), a thorough analysis of the interactive network was conducted using the cytoNCA plug-in to identify the important genes with high correlations. Finally, the transcriptomics data were subjected to gene set enrichment analysis (GSEA).

### Metabolomics and lipidomics data analysis

First, X (raw data preprocessed values) peaks were detected by quartile range denoising, and X metabolites/lipids were retained. The original data's missing values were then filled in with a minimum value of half. The data were further normalised using the total ion flow normalisation technique. The collected 3D data were imported into the SIMCA14.1 software package (V14.1, Sartorius Stedim Data Analytics AB, Umea, Sweden) for orthogonal partial least squares-discriminant analysis (OPLS-DA). The program then produced R2Y and Q2Y as the classification parameters, which indicated reliability, suitability, and statistical significance. Sevenfold cross-validation assessed the robustness of the model, which was then further validated by replacement testing. After 200 permutations, the intercept values of R2 and Q2 were found. A lower value of the Q2 intercept indicates good robustness of the model and that the risk of overfitting is low and reliable. The first principal component (VIP) of the significance of each variable in the forecast was calculated by OPLS-DA. First, the variable metabolites/lipids with VIP > 1 were selected. The remaining variables were subjected to Student's t test (*P* < 0.05), and then the differences between the two groups were eliminated. Additionally, metabolite pathway searches were conducted using commercial databases such as KEGG and MetaboAnalyst.

### Combined multiomics analysis

The KEGG pathway enrichment results were compared between the 3 omics groups to find the common enrichment pathways. Next, a correlation clustering analysis of transcriptomics and lipidomics was performed in R. The association between DELs and DEGs was calculated based on Spearman correlation analysis, and pairs with a *P* < 0.05 were chosen for network construction.

### HE and sirius red staining

Adipose tissue was removed from paraformaldehyde, dehydrated and embedded in wax, after which the wax blocks were placed in a microtome and sectioned at a thickness of 4 μm. The sections were deparaffinized and stained with haematoxylin and eosin (HE) (G1003, Servicebio, China) and Sirius red stain (G1018, Servicebio, China) to determine lipid droplet alterations and adipose tissue fibrosis, respectively. Sections stained with HE and Sirius red were examined with an ortho-optical Nikon Eclipse E100 microscope (Nikon, Tokyo, Japan). Images were captured using a Nikon DS-U3 imaging system (Nikon, Tokyo, Japan), and ImageJ software (NIH, USA) was used to measure lipid droplet sizes and the red area fraction of collagen fibres.

### Immunohistochemical staining

After the sections were deparaffinized, they were placed in a microwave oven with citric acid buffer for antigen retrieval (pH 6.0) and washed three times with phosphate buffered saline, and endogenous peroxidase with blocked with 3% hydrogen peroxide solution. The CD68 primary antibody (1:200; GB1131094, Servicebio, China) was diluted at a specific ratio and added dropwise to PBS before being added dropwise to the adipose tissue sections, which were uniformly covered with 3% BSA in a humidified chamber. The type of secondary antibody (1:200; G1213-100UL, Servicebio, China) (HRP-labelled) was matched to the primary antibody. The sections were incubated with freshly prepared DAB solution as a colour development agent before being observed under a microscope until a brownish yellow colour appeared in positive areas; the colour development time was adjusted accordingly. After the nuclei were stained with haematoxylin, the sections were dehydrated and sealed. Finally, images were obtained and examined in the same way as for Sirius red staining.

### Enzyme-linked immunosorbent assay (ELISA)

Four ELISA kits [Cer (Cat No. MM61454R2), S1P (Cat No. JYM1296Ra), IL-6 (Cat No. JYM0646Ra), TNF-α (Cat No. JYM0635Ra)] were used to quantify the levels of ceramide (Cer), sphingosine 1-phosphate (S1P), IL-6 and tumour necrosis factor-alpha (TNF-α) in adipose tissue. The levels of IL-6 and TNF-α were also quantified in plasma. All experimental procedures were performed according to the instructions provided with the ELISA kits.

### Real-time Quantitative PCR (RT‒qPCR)

SparkZol Reagent (Cat No. AC0101, SparkJade, China) was used to extract total RNA from adipose tissue. Then, total RNA was reverse-transcribed into cDNA using SPARKscript II All-in-one RT SuperMix for qPCR (with gDNA Eraser) (AG0305-B, SparkJade, China). A LightCycler 480 SYBR Green I Master (Roche, Germany) was then used to perform RT‒qPCR. 2^−ΔΔCT^ was used to calculate the relative expression of each target gene and normalised to β-actin [34]. The primer sequences (Sangon Biotech, Shanghai, China) are listed in Table 1.

**Table 1:** Primers sequence of RT-qPCR

Gene	Sequence(5’-3’)
β-actin	Forward 5’-AGCCATGTACGTAGCCATCC’ Reverse 5’-CTCTCAGCTGTGGTGGTGAA’
Col1	Forward 5’-TGGTCCTGCTGGCAAGAATGG’ Reverse 5’-TCTGTCACCTTGTTCGCCTGTC’
Col6	Forward 5’-CCCACGCAGAACAACCGAATTG’ Reverse 5’-TCCTTGATGCCCACAGAAACTACC’
Col14	Forward 5’-CCGTGACTTCAGTTCTCCAGACAG’ Reverse 5’-TCCAGGCACCATAACCACTTCTTC’
Tgf-β	Forward 5’-CAAGTCAACTGTGGAGCAAC’ Reverse 5’-AACCCAGGTCCTTCCTAAAG’
Smad2	Forward 5’-CAAGGCGATCGAGAACTGCG’ Reverse 5’-GCCGTCTACAGTGAGTGAGG’
Ccl5	Forward 5’-ACACCACTCCCTGCTGCTTTG’ Reverse 5’-TCTCTGGGTTGGCACACACTTG’
Mdk	Forward 5’-TTCTAGCCCTTGTTGCCCTCTTG’ Reverse 5’-ACACTCGCTGCCCTTCTTCAC’
Mmp14	Forward 5’-TGAGGAGGAGACGGAGGTGATC’ Reverse 5’-CAGTACCAGGAGCAGCAGCAG’

### Western blot analysis

A MinuteTM Animal Adipose Tissue Protein Extraction Kit (Cat No. AT-022, Invent, Minnesota, USA) was used to extract protein from adipose tissue. A bicinchoninic acid (BCA) kit (Cat No. CW0014S, Cwbio, Jiangsu, China) was used to assess the protein concentration, which was adjusted to 2 µg/µL. Next, 15 µl of diluted protein from each sample was isolated using a One-Step PAGE Gel Fast Preparation Kit (10%) (Cat No. E303-01, Vazyme, Nanjing, China) and then transferred to PVDF membranes (Cat No. WGPVDF45, Millipore, Massachusetts, USA) for immunoblotting. The membranes were then incubated in 5% skim milk at 25 °C for 1 h. Subsequently, the membranes were washed and incubated with the following primary antibodies overnight at 4 °C: β-actin (Cat No. 20536–1-AP, Proteintech, Wuhan, China), sphingosine kinase (SPHK) (1:1000, Cat No. 10670–1-AP, Proteintech, Wuhan, China), sphingosine 1-phosphate receptor 1 (S1PR1) (1:1000, Cat No. 55133–1-AP, Proteintech, Wuhan, China) and sphingosine 1-phosphate receptor 2 (S1PR2) (1:1000, Cat No. ab235919, Abcam, Cambridge, United Kingdom). The membranes were washed and incubated with species-specific secondary antibodies conjugated to HRP. An enhanced chemiluminescence (ECL) detection kit (Cat No. PK10002; Proteintech; Wuhan; China) was used to visualise the protein bands. Finally, FluorChem Q software v3.4 (ProteinSimple; California; USA) was used to perform densitometric analysis and calculate the protein content.

### Statistical analysis

Statistical analyses were performed using GraphPad Prism software (v. 9) and RStudio. The results are expressed as the mean ± SD. Univariate statistical analyses were performed using Student's t test to determine statistical significance. *P* < 0.05 was considered a statistically significant difference.

## Results

### MCT injection severely reduced ventricular function and induced body weight loss

**Fig. 2:**
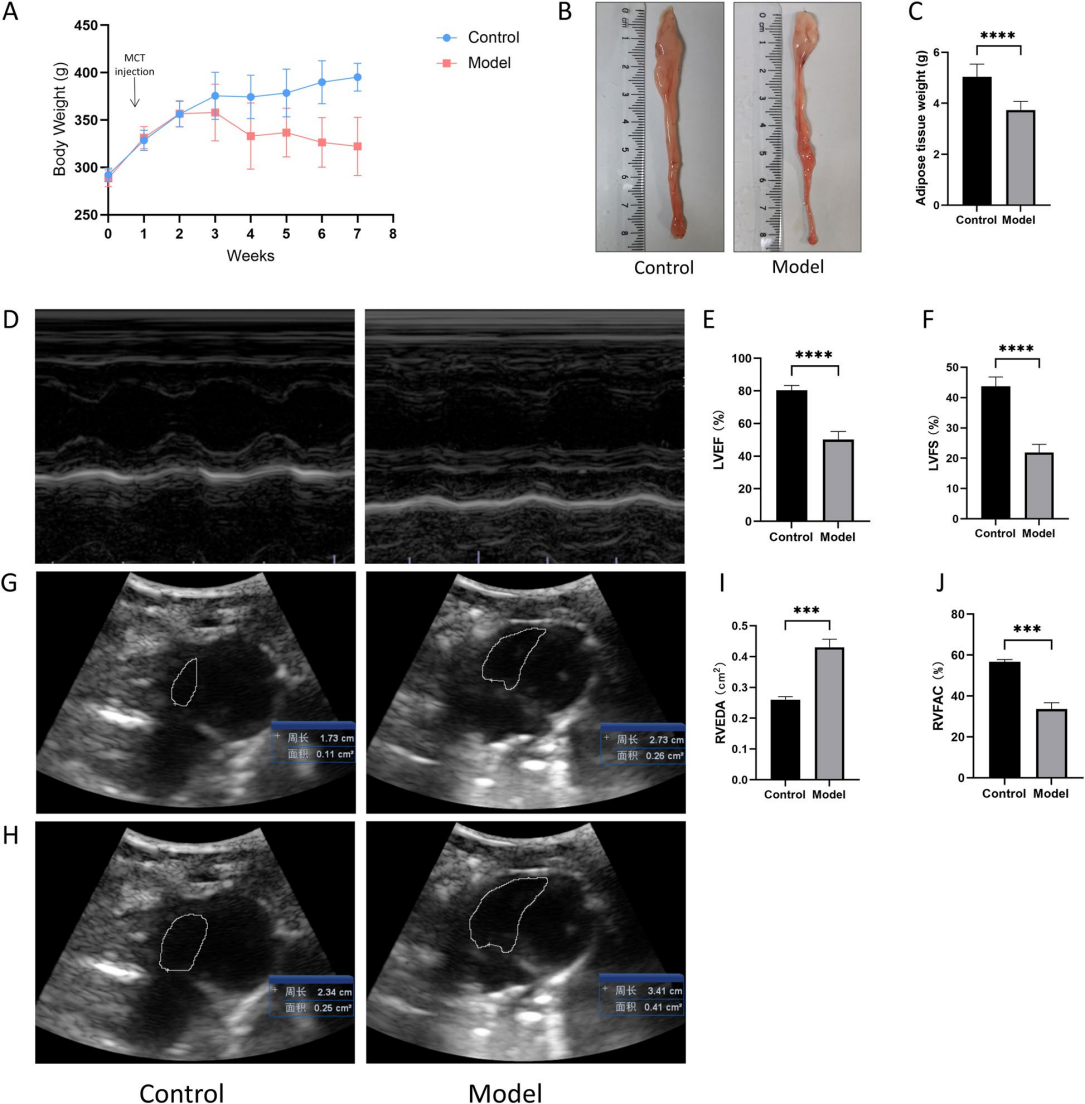
Body weight and ventricular function of rats. **A** Changes in body weight through the duration of the 6-week study. **B** Original gross images of the adipose tissue. **C** Adipose tissue weight. **D** Representative images of echocardiography reflecting left ventricular function. **E** Left ventricular ejection fraction (LVEF). **F** Left ventricular fractional shortening (LVFS). **G**, **H** Representative images of echocardiography reflecting right ventricular function. **I** Right ventricular end-diastolic area (RVEDA). **J** Right ventricular fractional area change (RVFAC). Data are mean ± SD, *n* = 6–10. ***P < 0.001, ****P < 0.0001 vs. the control group

Two weeks after receiving MCT injections, body weight in the model group began to decrease compared to that in the control group. Rats in the model group had significantly lower body weights by the end of the sixth week than rats in the control group (*P* < 0.01; Fig. 2A). Adipose tissue in the model group was significantly atrophied, and the total weight of adipose tissue was reduced (*P* < 0.01; Fig. 2B, C). In addition, ultrasound cardiography demonstrated a substantial decrease in LVEF, LVFS, RVEDA, and RVFAC in the model group (*P* < 0.01; Fig. 2D-J). This suggests that the injection of MCT induced HF and cachexia.

### Transcriptomics revealed that DEGs were primarily involved in inflammatory and fibrotic processes

**Fig. 3:**
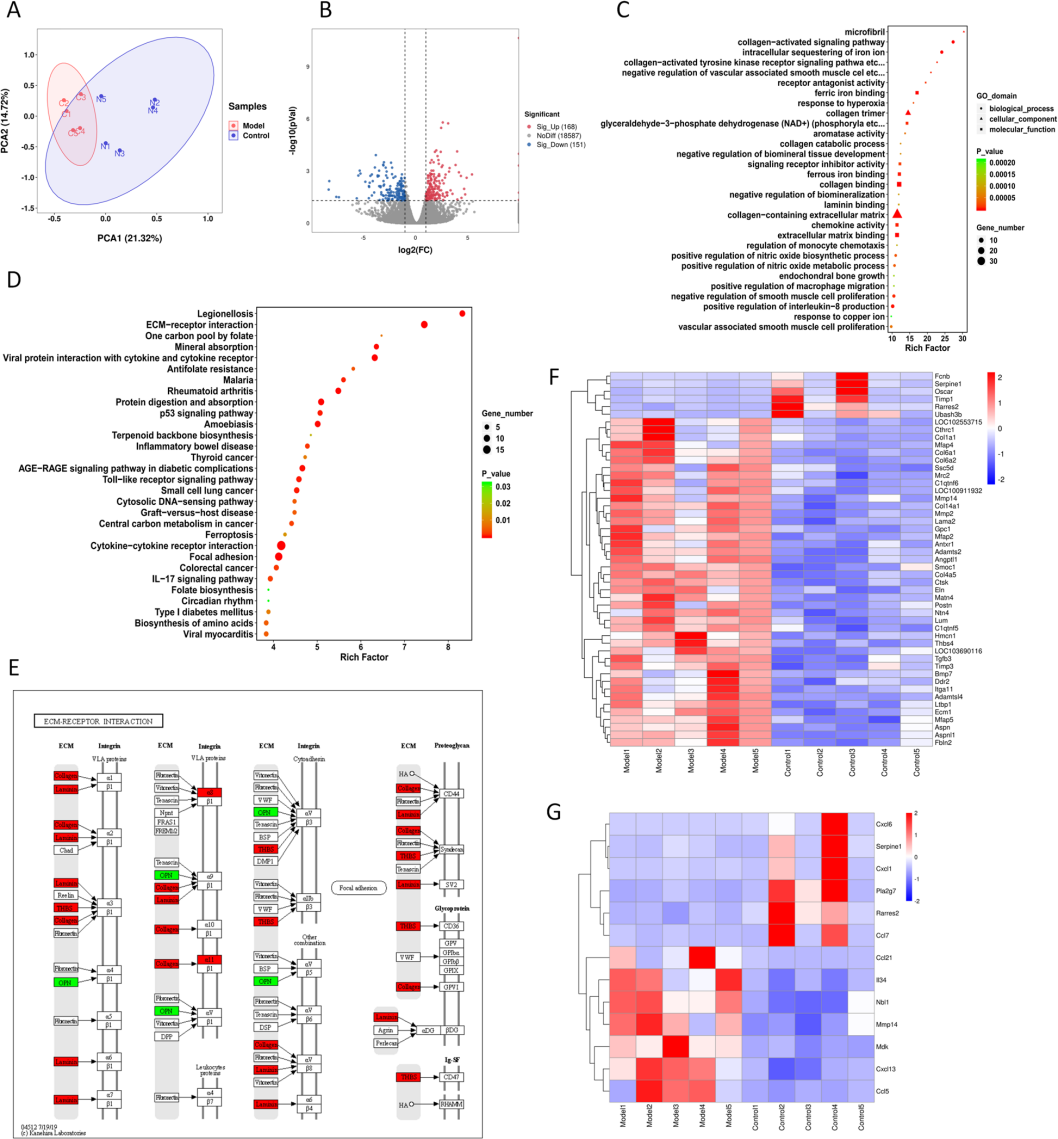
Transcriptomics data analysis. **A** PCA analysis plot. The horizontal and vertical coordinates represent the top two principal components and their contribution to the sample ranking. **B** Volcano plot of DEGs. **C** Results of GO enrichment of DEGs. **D** Results of KEGG enrichment of DEGs. **E** Demonstration of DEGs in the ECM-receptor interaction pathway. KO nodes with upregulated genes are shown in red, and KO nodes with downregulated genes are shown in green. **F** Heat map of DEGs associated with fibrosis. **G** Heat map of DEGs associated with inflammation

To elucidate the pathogenesis of adipose tissue dysfunction in cardiac cachexia, we performed transcriptome sequencing analysis. The relationship and variance between the samples were evaluated using principal component analysis (PCA), which demonstrated a distinct difference between the control and model groups (Fig. 3A). The DEG screening criteria were log2(FC) > 1 and *Q* value < 0.05. A volcano plot was used to visualise the DEGs (Fig. 3B) after 320 DEGs were selected, of which 151 were downregulated and 168 were upregulated.

GO enrichment analysis was performed on the terms in the GO database, and the top 30 GO terms that were significantly enriched in the DEGs were screened using a threshold of *Q* value < 0.05 (Fig. 3C). Among them, the largest number of terms and the most significant enrichment levels were found for biological processes related to fibrosis, such as microfibrils, collagen-activated signalling pathway, collagen binding, collagen catabolic process, collagen-activated tyrosine kinase receptor signalling pathway, extracellular matrix binding, and collagen-containing extracellular matrix. The next aspect was inflammation, such as positive regulation of macrophage migration and regulation of monocyte chemotaxis. As shown in Table 2, the expression levels of genes associated with fibrosis, such as Col1, Col4, Col6, Col14, Ecm1, Postn, and Eln, were increased. Similarly, the expression levels of genes associated with monocyte/macrophage migration, including Ccl5, Nbl1, Mdk, and Mmp14, were also upregulated.

**Table 2:** GO terms associated with fibrosis and inflammation in transcriptomics analysis

Class	GO term	Gene up	Gene down	Rich factor	*P*value	Qvalue
Fibrotic process	microfibril	Mfap5, Ltbp1, Mfap4, Mfap2		30.39	2.23E-06	1.70E-04
	collagen-activated signaling pathway	Col1a1, Col4a5, Ddr2, Itga11	Ubash3b, Oscar	27.35	3.64E-08	8.45E-06
	collagen-activated tyrosine kinase receptor signaling pathway	Col1a1, Col4a5, Ddr2	Ubash3b	22.80	6.66E-06	3.52E-04
	collagen trimer	Col4a5, C1qtnf5, Col6a1, Cthrc1, C1qtnf6, Col1a1, Col6a2, Col14a1	Fcnb	14.55	1.36E-09	7.10E-07
	collagen catabolic process	Mrc2, Mmp2, Ctsk, Mmp14		12.43	7.70E-05	2.05E-03
	collagen binding	Aspnl1, Aspn, Antxr1, Mrc2, Ctsk, Col6a2, Itga11, Lum, Ddr2, Col6a1		12.00	8.02E-09	3.05E-06
	collagen-containing extracellular matrix	Ssc5d, Lum, Col6a2, Gpc1, Eln, Thbs4, Ntn4, Col1a1, LOC102553715, Adamtsl4, Hmcn1, Tgfb3, Col14a1, LOC103690116, Col6a1, Timp3, Ltbp1, Smoc1, Adamts2, Mfap4, Angptl1, Lama2, Ecm1, Matn4, Fbln2, Col4a5, Bmp7, Postn	Timp1, Serpine1, Fcnb, Rarres2	11.46	8.21E-23	1.14E-19
	extracellular matrix binding	Ecm1, Gpc1, Ssc5d, Smoc1, Ntn4, Fbln2, Eln, LOC100911932		11.40	2.58E-07	3.72E-05
Inflammatory process	chemokine activity	Cxcl13, Ccl5, Ccl21	Cxcl1, Ccl7, Cxcl6	11.40	5.28E-06	2.94E-04
	regulation of monocyte chemotaxis	Ccl5, Nbl1	Serpine1, Pla2g7	11.40	1.11E-04	2.54E-03
	positive regulation of macrophage migration	Mdk, Mmp14, Il34	Rarres2	10.52	1.55E-04	3.26E-03

Consistent with the KEGG enrichment analysis, DEGs were linked to ECM receptor interactions and cytokine receptor interactions (Fig. 3D); 11 genes, including the upregulated genes Col1a1, Col4a5, Col6a1, Col6a2, Itga8, Itga11, Lama2, Thbs3, Thbs4 and the downregulated gene Spp1, were enriched for ECM-receptor interactions (*Q*-value = 3.74E-05). The combined GO and KEGG results indicated that DEGs in adipose tissue were mainly associated with fibrosis and inflammation. The heatmap showed significant differences in DEGs related to fibrosis and inflammation (Fig. 3F, G), suggesting that these DEG-related pathways and biological processes may perform significant roles in the homeostatic imbalance of adipose tissue in cardiac cachexia.

### Inflammation-associated DEGs closely interact with fibrosis-associated DEGs

**Fig. 4:**
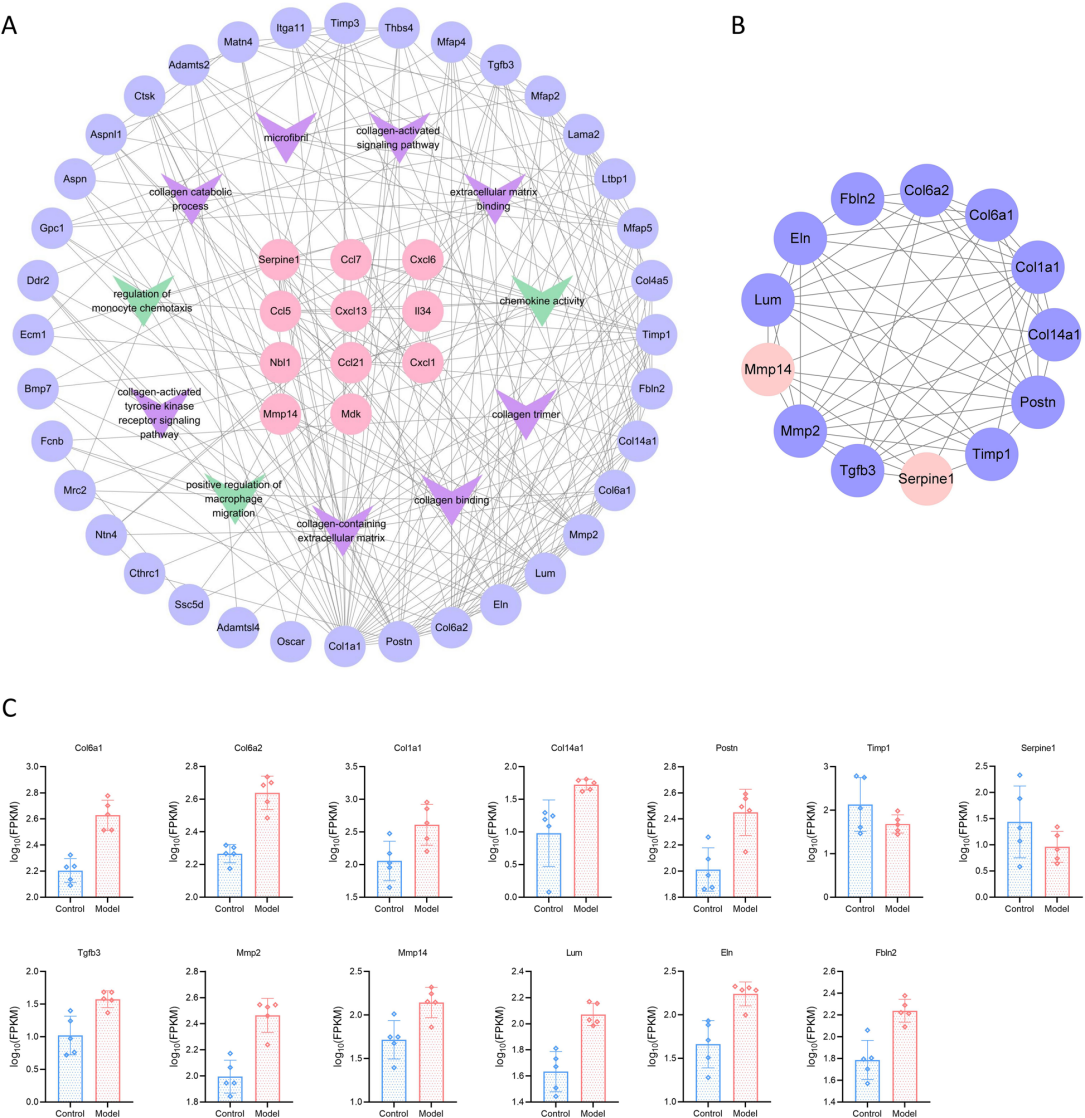
Analysis of the interaction between inflammation-associated DEGs and fibrosis-associated DEGs. **A** Interactive network diagram of inflammation-associated DEGs and fibrosis-associated DEGs. **B** Interactive network diagram of 13 strongly interacting DEGs. **C** Expression of strongly interacting DEGs in adipose tissue

DEGs associated with inflammation and fibrosis were incorporated into the STRING database for interactive network analysis. The data output was imported into Cytoscape software for visualisation (Fig. 4A). The results showed close interactions between inflammation-associated DEGs and fibrosis-associated DEGs. After screening by centrality analysis using the cytoNCA plugin, 13 strongly interacting DEGs were obtained: Col1a1, Col14a1, Col6a1, Col6a2, Timp1, Mmp2, Postn, Lum, Serpine1, Tgfb3, Mmp14, Fbln2, and Eln (Fig. 4B). Figure 4C shows the expression of the aforementioned genes.

### The expression of monocyte chemotaxis/macrophage migration and fibrosis gene sets was upregulated in cardiac cachexia

**Fig. 5:**
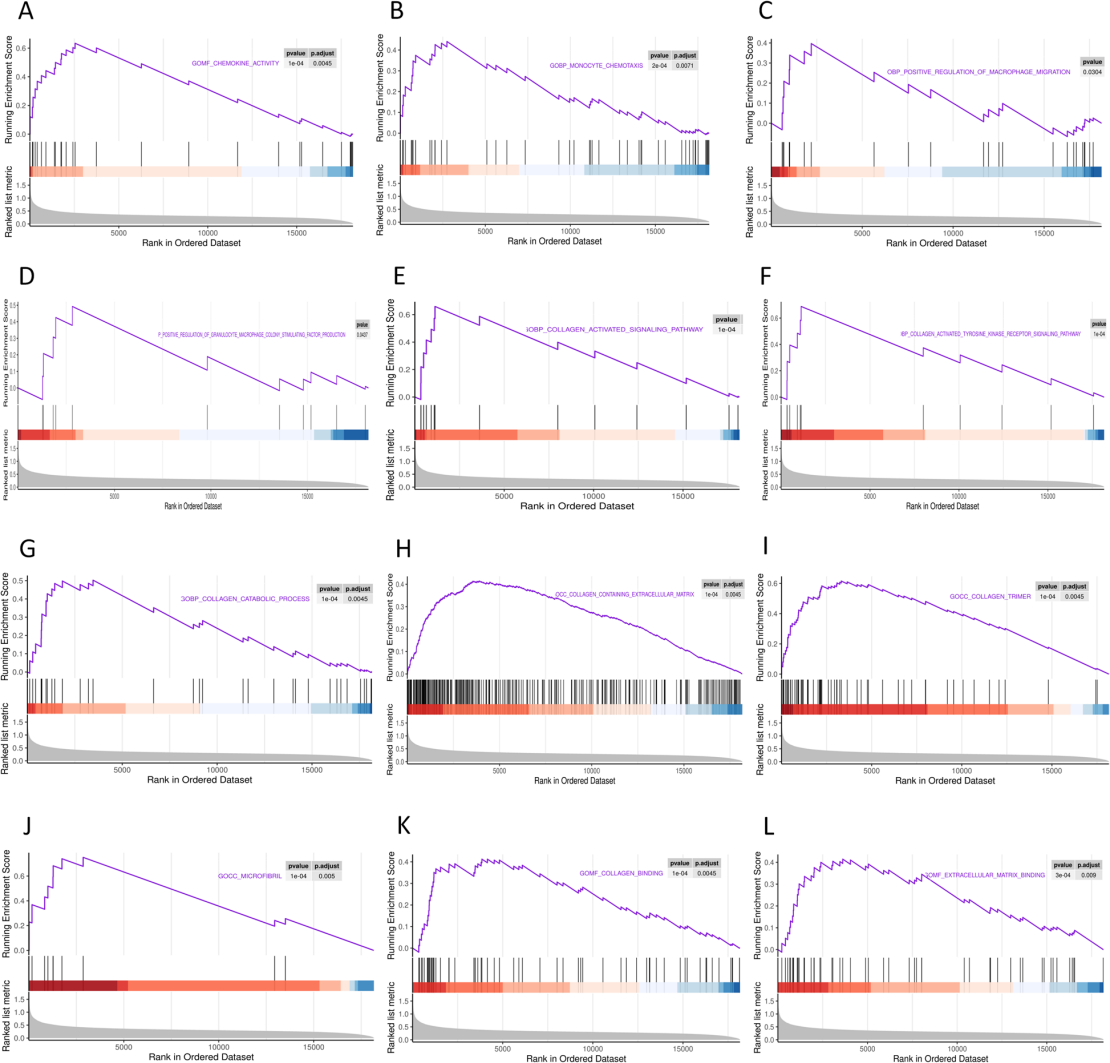
Gene set enrichment analysis. **A** GSEA of ‘Chemokine activity’. **B** GSEA of ‘Monocyte chemotaxis’. **C** GSEA of ‘Positive regulation of macrophage migration’. **D** GSEA of ‘Positive regulation of granulocyte macrophage colony stimulating factor production’. **E** GSEA of ‘Collagen activated signalling pathway’. **F** GSEA of ‘Collagen activated tyrosine kinase receptor signalling pathway’. **G** GSEA of ‘Collagen catabolic process’. **H** GSEA of ‘Collagen containing extracellular matrix’. **I** GSEA of ‘Collagen trimer’. **J** GSEA of ‘Microfibril’. **K** GSEA of ‘Collagen binding’. **L** GSEA of ‘Extracellular matrix binding’

GSEA can show significant functional differences caused by the accumulation of trace changes in a batch of genes. GO analysis showed that the gene sets for inflammation were selected as ‘chemokine activity’, ‘monocyte chemotaxis’, ‘positive regulation of macrophage migration’, and ‘positive regulation of granulocyte macrophage colony-stimulating factor production’. The results showed that the gene sets for monocyte chemotaxis, macrophage migration, and aggregation were significantly upregulated (*P* < 0.05; Fig. 5A-D). Similarly, the gene sets associated with fibrosis, including ‘collagen activated signalling pathway’, ‘collagen catabolic process’, ‘collagen containing extracellular matrix’, ‘microfibril’, ‘collagen trimer’, ‘collagen activated tyrosine kinase receptor signalling pathway’, ‘collagen binding’, and ‘extracellular matrix binding’, also showed high levels of expression (*P* < 0.05; Fig. 5E-L). This evidence suggests that adipose tissue remodelling in cardiac cachexia is linked to inflammatory and fibrotic alterations.

### Metabolomics identified the sphingolipid signalling pathway as a critical mechanism in cardiac cachexia

**Fig. 6:**
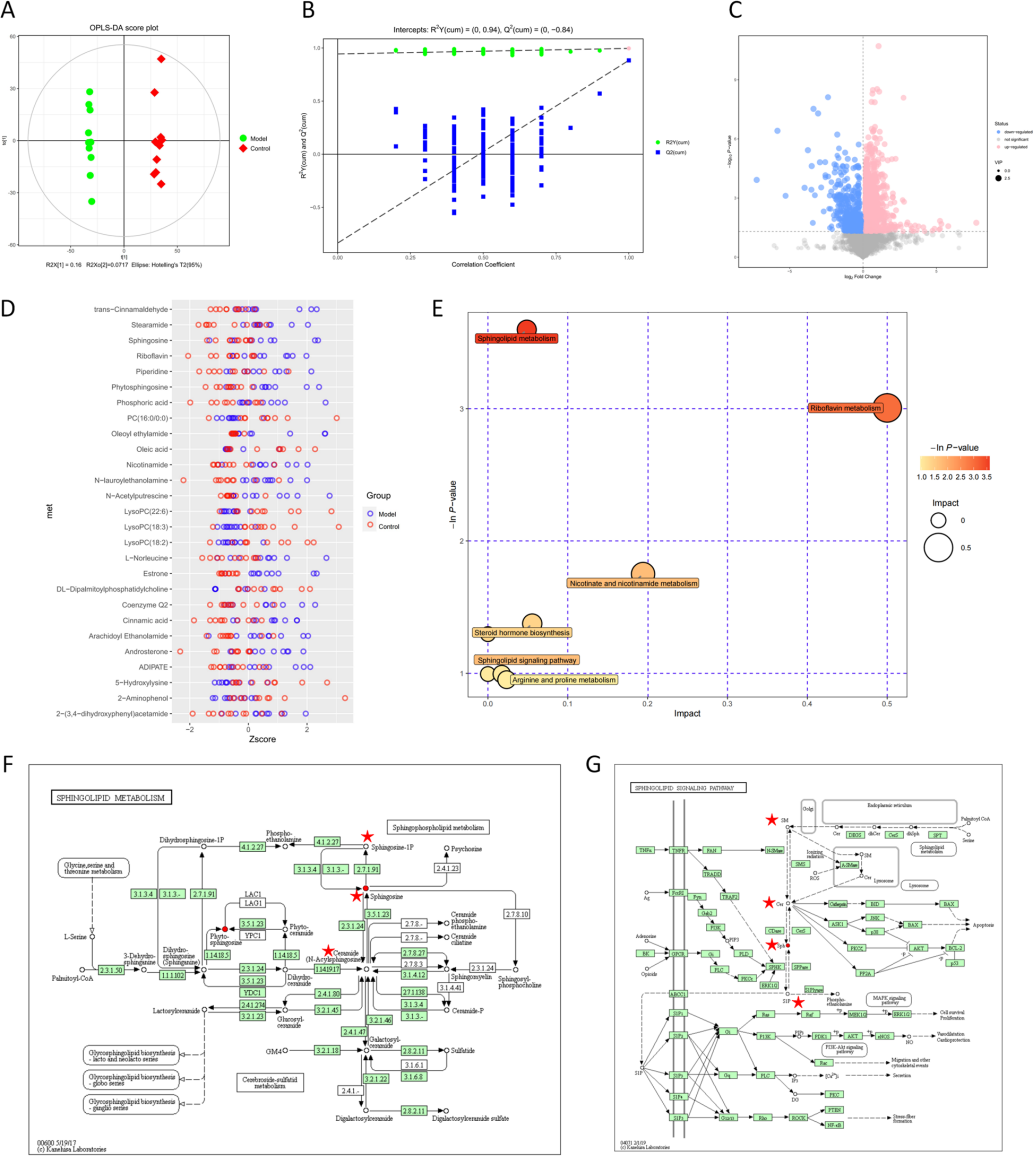
Metabolomics analysis. **A** Scatter plot of the scores from the OPLS-DA model. **B** Results of the replacement test of the OPLS-DA model. **C** Volcano plot of DEMs. **D** Z-score plot of DEMs. **E** Diagram of the metabolic pathway analysis of DEMs. **F** Diagram of the process of sphingolipid metabolism. **G** Diagram of the sphingolipid signalling pathway

The raw mass spectrometry files were imported into Compound Discoverer 3.1 (CD) software for the characterisation and quantification of metabolites. The metabolites were then subjected to multivariate statistical analysis. The two groups exhibited a statistically significant difference according to the OPLS-DA score plot (Fig. 6A). To perform the permutation test and acquire the R2 and Q2 of the random model, the order of the categorical variable Y was randomly changed, and the matching OPLS-DA model was built numerous times (*n* = 200). The OPLS-DA model's permutation test results (Fig. 6B) showed that the original model had a decent level of robustness [35]. Metabolite expression was visualised as volcano plots (Fig. 6C) with filtering conditions of VIP > 1 and P 0.05, and 27 DEMs were obtained. The z score plot showed the relative levels of the 27 DEMs in each sample (Fig. 6D). After obtaining the matching data for the DEMs from reliable databases, metabolic pathway studies were performed. The results are displayed in Table 3. The pathways that highlighted the importance after the combined enrichment analysis and topological analysis were labelled (Fig. 6E). As shown in Fig. 6E, sphingolipid metabolism was the most critical of the metabolic pathways, and the sphingolipid signalling pathway was similarly enriched. A diagram of the sphingolipid metabolic process and the sphingolipid signalling pathway is shown in Fig. 6F and G. Among them, Cer and S1P are the core metabolites of the sphingolipid signalling pathway.

**Table 3:** Metabolic pathways of 27 DEMs in metabolomics analysis

Pathway	Total	Hits	Raw p	-Ln(p)	Holm adjust	FDR	Impact	Hits Cpd
Sphingolipid metabolism	21	2	0.027361	3.5986	1	1	0.04868	Sphingosine; Phytosphingosine
Riboflavin metabolism	4	1	0.049469	3.0064	1	1	0.5	Riboflavin
Nicotinate and nicotinamide metabolism	15	1	0.17383	1.7497	1	1	0.1943	Nicotinamide
Steroid hormone biosynthesis	77	2	0.25244	1.3766	1	1	0.05551	Androsterone; Estrone
Lysine degradation	25	1	0.27337	1.2969	1	1	0	L-Hydroxylysine
Biosynthesis of unsaturated fatty acids	36	1	0.36971	0.99505	1	1	0	(9Z)-Octadecenoic acid
Glycerophospholipid metabolism	36	1	0.36971	0.99505	1	1	0.01736	1-Acyl-sn-glycero-3-phosphocholine
Arginine and proline metabolism	38	1	0.38587	0.95227	1	1	0.02346	N-Acetylputrescine
Sphingolipid signaling pathway	15	1	0.17383	1.7497	1	1	0	Sphingosine

### Lipidomics identified increased levels of Cer and S1P in adipose tissue in cardiac cachexia

**Fig. 7:**
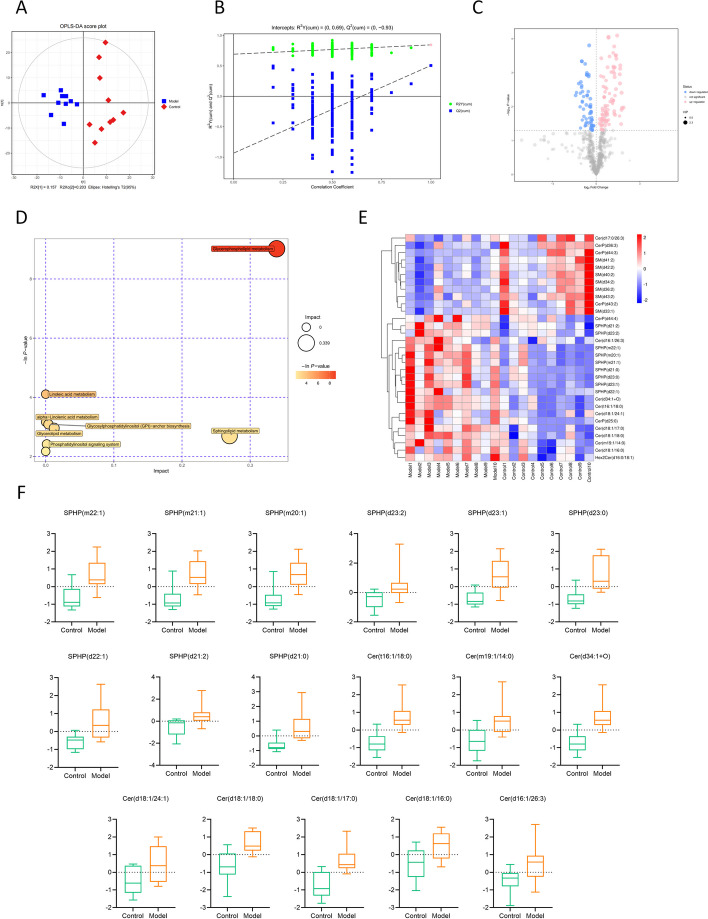
Lipidomics analysis. **A** Scatter plot of the scores from the OPLS-DA model. **B** Results of the displacement test of the OPLS-DA model. **C** Volcano plot of DELs. **D** Z-score plot of DELs. **E** Heat map of differentially expressed sphingolipids. **F** Box plots of the relative expressions of S1P and Cer

The raw lipidome files obtained by mass spectrometry were imported into Lipid Search (Thermo Corporation) software for spectral processing and database searching to characterise and quantify lipids. The OPLS-DA results (Fig. 7A) showed a high level of intragroup aggregation, and all samples fell within the 95% confidence range. This indicates that the two groups of samples were substantially distinct from each other. The permutation test (Fig. 7B) indicated that the original model was robust. DELs with a *P* < 0.05 were selected, and the importance of the VIP > 1. A total of 143 DELs were obtained (Fig. 7C), of which 85 were upregulated and 58 were downregulated. Metabolic pathway analysis revealed that the seven metabolic pathways with *P* < 0.1 were considered the most relevant metabolic pathways (Fig. 7D). As expected, the metabolic pathway enrichment results showed enrichment in the sphingolipid metabolic pathway (*P* < 0.05), reinforcing the fact that sphingolipid metabolism plays a significant part in adipose tissue remodelling in malignant disease. The differentially expressed sphingolipids are shown in the heatmap (Fig. 7E). Among these sphingolipids, S1P (SPHP) and Cer expression were predominantly upregulated. The relative expression levels of S1P and Cer are shown in Fig. 7F.

### Combined multiomics analysis suggested that the sphingolipid metabolic pathway was associated with inflammatory-fibrotic changes in adipose tissue

**Fig. 8:**
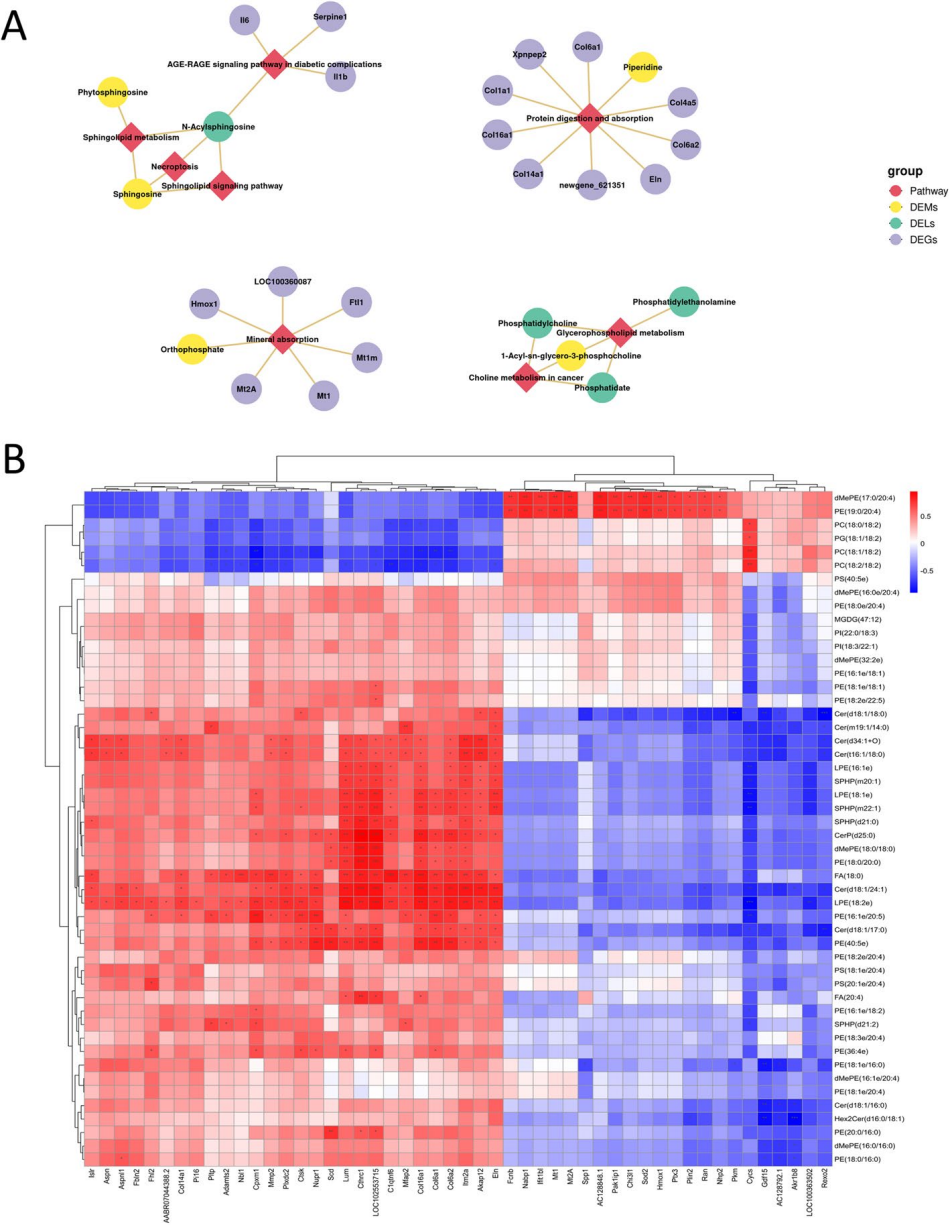
Combined multi-omics analysis. **A** DEGs-DEMs-DELs shared enriched KEGG pathway network map. **B** Heatmap of correlation clustering of DEGs and DELs. *P < 0.05, **P < 0.01, ***P < 0.001

DEGs, DEMs and DELs were enriched in some common KEGG pathways. Notably, phytosphingosine and sphingosine (Sph) were enriched with Cer (N-acylsphingosine) in the sphingolipid metabolic pathway. In addition, DEGs (Col1, Col4, Col6, Col14, Col16, etc.) and piperidine were enriched in the protein digestion and absorption pathways (Fig. 8A). This further highlights the critical role of the sphingolipid metabolic pathway and extracellular matrix remodelling in adipose tissue dysfunction, and this study performed a correlation analysis of DEGs and DELs. The correlation clustering heatmap (Fig. 8B) shows the correlation of the top 50 DEGs with the top 50 DELs, and Cer and S1P were positively correlated with inflammation/fibrosis-associated DEGs.

**Fig. 9:**
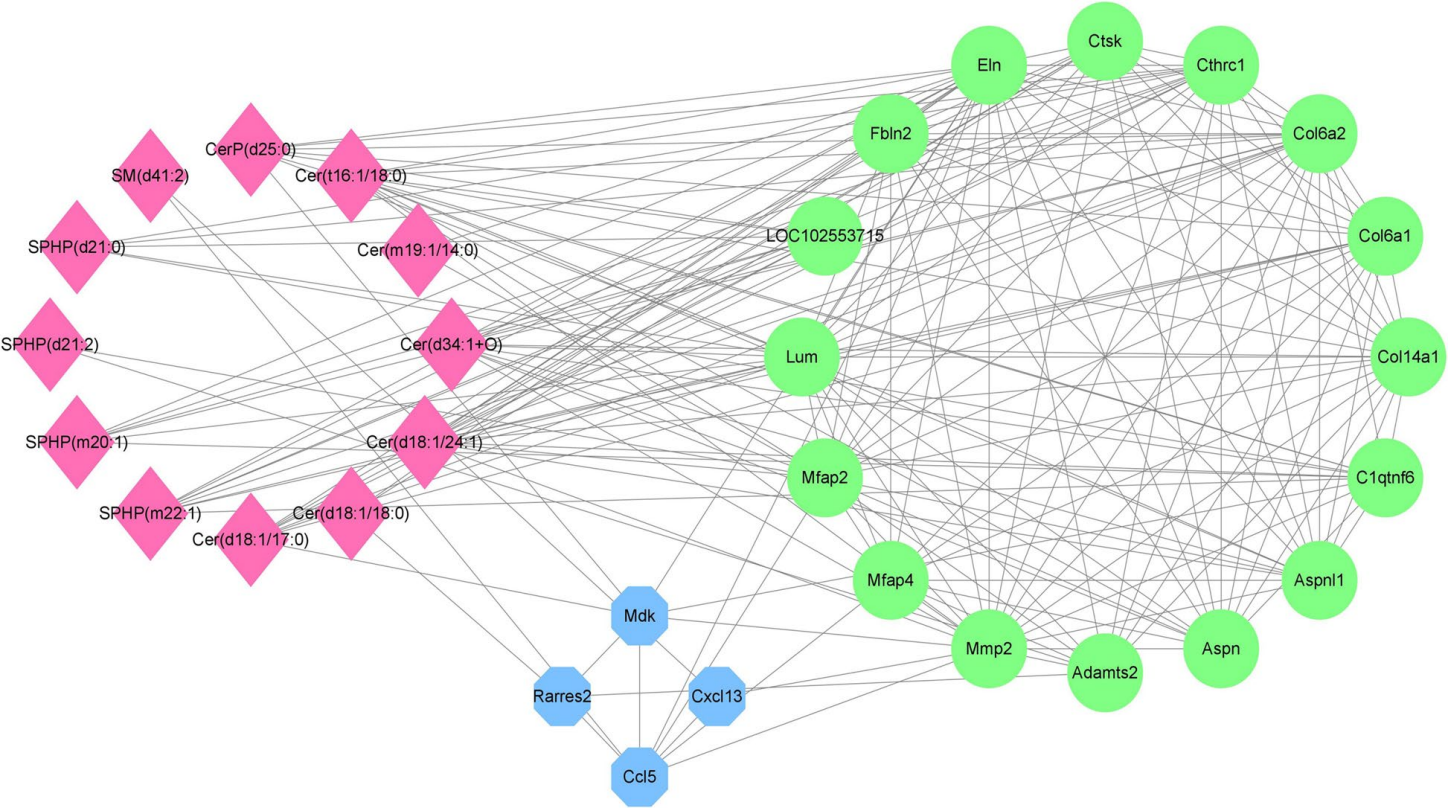
Network diagram of the correlation between inflammation-associated DEGs and fibrosis-associated DEGs, as well as differentially expressed sphingolipids

Figure 9 shows the network correlation between differentially expressed sphingolipids, inflammation-associated DEGs and fibrosis-associated DEGs. S1P was associated with monocyte/macrophage migration and extracellular matrix remodelling, and the latter two were interrelated. Integrated lipidomics and transcriptomics analysis provides potential implications for understanding the mechanisms of cachexia-induced adipose tissue remodelling.

### Adipose tissue had increased levels of sphingolipids and exhibited macrophage infiltration and fibrosis during cardiac cachexia

**Fig. 10:**
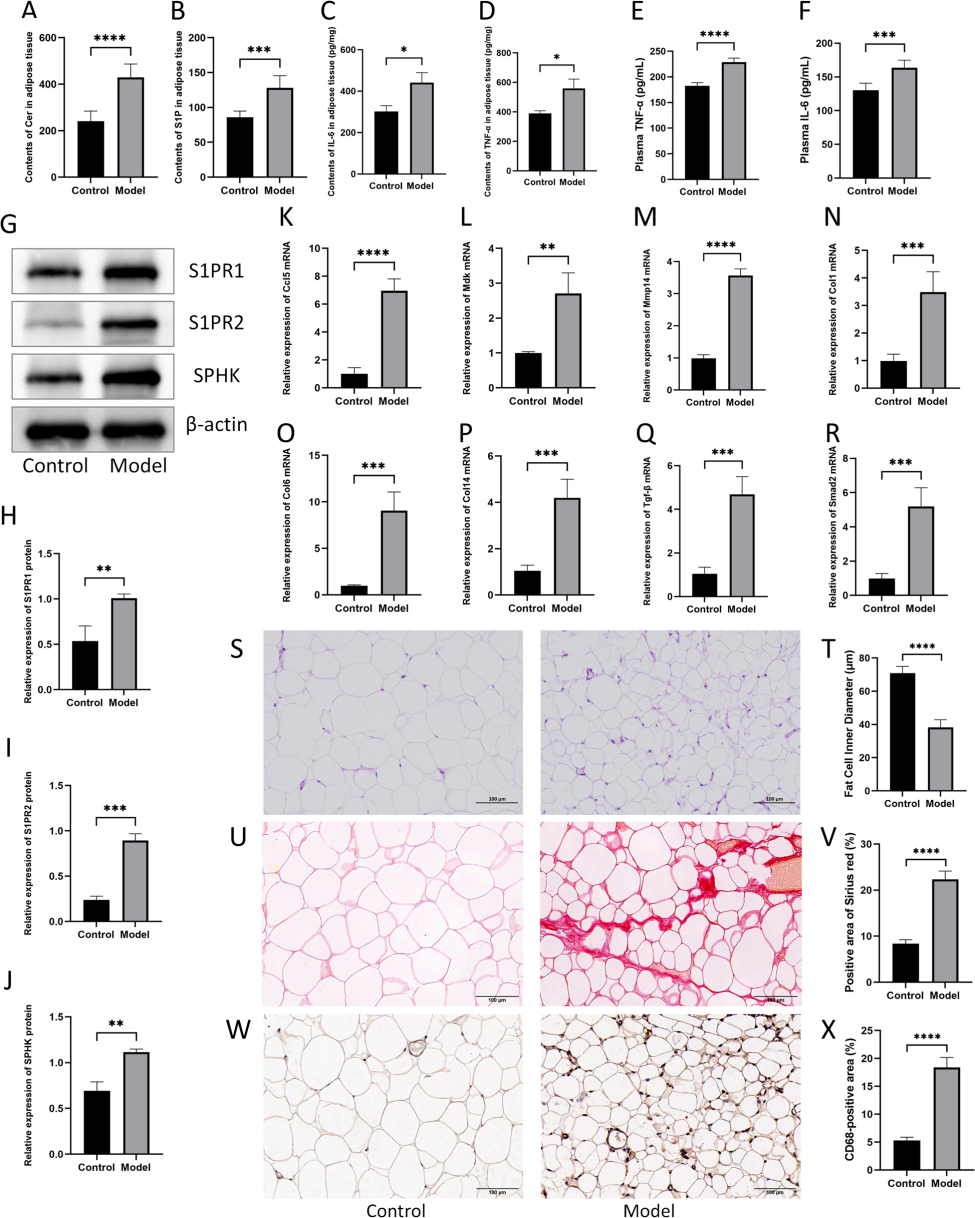
Experimental verification. **A**, **B** Contents of Cer and S1P in adipose tissue. **C**, **D** Contents of IL-6 and TNF-α in adipose tissue. **E**, **F** Contents of IL-6 and TNF-α in plasma. **G**-**J** Protein expression of S1PR1, S1PR2, and SPHK measured by Western blot in adipose tissue. **K**-**M** mRNA expression of monocyte/macrophage chemotaxis genes, including Ccl5, Mdk and Mmp14. **N**-**P** mRNA expression of representative fibrosis- associated genes, including Col1, Col6, and Col14. **Q**, **R** mRNA expression of Tgf-β/Smad signalling pathway genes, including Tgf-β and Smad2. **S, T** Representative images of HE staining and fat cell inner diameter of adipose tissue. **U**, **V** Representative images of Sirius scarlet staining and positive area of adipose tissue. **W**, **X** Representative images of immunostaining and positive areas of CD68 in adipose tissue. Scale bar = 100 μm. Data are mean ± SD, *n* = 6. **P < 0.01, ***P < 0.001, ****P < 0.0001 vs. the control group

Some crucial indications were experimentally evaluated to validate the study results. Most notably, the ELISA data proved that the levels of Cer and S1P were higher in cardiac cachexia adipose tissue than in normal rats (*P* < 0.001; Fig. 10A, B). Similarly, the levels of the inflammatory cytokines IL-6 and TNF-α were increased in adipose tissue (*P* < 0.05; Fig. 10C, D) and plasma (*P* < 0.001; Fig. 10E, F), suggesting systemic inflammation in cardiac cachexia. S1P is the major biologically active sphingolipid that is produced by the phosphorylation of Sph by SPHK and acts through S1PRs [36, 37]. The generation of cytokines and chemokines, as well as fibrosis, are biological processes in which the SPHK/S1P/S1PR axis is engaged [38, 39]. In the adipose tissue of the model group, western blot analysis showed increased protein expression of SPHK, S1PR1, and S1PR2 (*P* < 0.01; Fig. 10G-J), indicating that the SPHK/S1P/S1PR axis was activated in cardiac cachexia. Furthermore, RT‒qPCR proved that the relative expression of monocyte chemotaxis/macrophage migration genes (Ccl5, Mdk, Mmp14) and fibrosis genes (Col1, Col6, Col14) was upregulated in cardiac cachexia (*P* < 0.01; Fig. 10K-P). A previous study demonstrated that the fibrotic response in adipose tissue during cancer-induced cachexia was triggered by inflammation, which activated fibroblasts and promoted extracellular matrix deposition through Tgf-β/Smad signalling. In this study, the transcriptomics results revealed increased expression of the Tgf-β gene. RT‒qPCR further demonstrated increased expression of the Tgf-β and Smad2 genes in cardiac cachexia adipose tissue (*P* < 0.001; Fig. 10Q, R), suggesting that Tgf-β/Smad signalling was activated. In cachexia, adipocyte size is decreased, and tissue matrix fibrosis is significantly increased, which is known as adipose tissue remodelling [40]. HE staining of adipose tissue droplets revealed that the inner diameter of fat cells in cachexia adipose tissue was smaller than that in non-cachexia adipose tissue (*P* < 0.01; Fig. 10S, T). Sirius red staining showed intense staining of the adipose extracellular matrix in cachectic rats (*P* < 0.01; Fig. 10U, V), indicating a significant increase in collagen fibril content in adipose tissue. This study also showed an increase in macrophages in the model group, characterised by increased expression of the marker CD68 (*P* < 0.01; Fig. 10W, X). Histological staining confirmed significant fibrotic changes and macrophage infiltration in adipose tissue in cardiac cachexia.

## Discussion

An important indicator of adipose tissue dysfunction is remodelling [41]. Since adipose tissue plays a significant role in homeostasis associated with energy metabolism [42], an imbalance in adipose tissue homeostasis in cachexia can cause dysfunction in lipid synthesis/decomposition in adipose tissue, which can lead to an imbalance in systemic energy metabolism and ectopic lipid deposition [43]. Therefore, effectively reducing adipose tissue dysfunction by restoring the homeostatic balance of adipose tissue would improve metabolic disorders in cachexia and improve the bottleneck associated with cachexia treatment.

This study is the first to combine transcriptomics, metabolomics and lipidomics analysis of adipose tissue samples in cardiac cachexia. The genes involved in fibrosis and monocyte/macrophage migration were upregulated in cardiac cachexia, as shown by transcriptomics analysis. Sirius red staining and CD68 staining showed increased collagen fibres and increased numbers of CD68-positive macrophages in cardiac cachexia adipose tissue. Consistent with previous studies of cancer-induced cachexia, this study confirmed the presence of fibrosis and macrophage infiltration in cardiac cachexia adipose tissue, leading to adipose tissue remodelling. In addition, metabolomics and lipidomics identified sphingolipid metabolic pathways as a major mechanism of cardiac malignancy, which was mainly characterised by increased levels of sphingolipids such as Cer and S1P. Network analysis of DEGs and DELs revealed a correlation between Cer/S1P and monocyte chemotaxis/macrophage migration genes, as well as fibrosis genes. This suggests that high levels of sphingolipids in cardiac cachexia adipose tissue may be associated with inflammatory and fibrotic changes.

The ECM serves as a crucial repository for collagen, growth factors, fibronectin, and metalloproteinases. The balance between adipocytes and the ECM is essential for the proper function of adipose tissue [44, 45]. In healthy adipose tissue, proper remodelling of the ECM provides a favourable microenvironment for the expansion of adipose tissue and maintains the normal architecture of adipose tissue and interadipocyte communication [46]. However, excessive accumulation of ECM can cause fibrosis in adipose tissue, which hinders the expansion of adipocytes, reduces the function of lipid storage, causes lipid spillage and interferes with normal signalling communication between adipocytes, which is an important pathological basis for adipose tissue dysfunction and causes metabolic disorders [46]. Under normal conditions, type VI collagen (COL6) is expressed more specifically in adipose tissue than in the liver, muscle, heart, and pancreas, and a lack of COL6 causes uncontrolled adipocyte growth in Col6 KO ob/ob mice [47].

Similarly, clinical investigations have revealed that individuals diagnosed with gastric cancer and cachexia exhibit fibrosis in subcutaneous adipose tissue. This fibrosis is characterised by increased levels of collagen types I, III, and VI, as well as fibronectin (Fn), within subcutaneous adipose tissue. Additionally, these individuals exhibit an increased number of myofibroblasts, which are responsible for synthesising extracellular matrix proteins around adipocytes. These findings are in contrast to the state of patients without concurrent cachexia. Furthermore, the expression of the TGF-β/SMAD signalling pathway, which is implicated in fibrosis, is also increased in individuals with gastric cancer and cachexia [19]. Adipose tissue dysfunction is suggested to be associated with fibrosis. Therefore, it is reasonable to speculate that blocking adipose tissue fibrosis would improve cachexia-associated metabolic disorders. The occurrence of inflammation is attributed to an increase in the accumulation of macrophages within adipose tissue, leading to an increase in the release of TNF-α, interleukins, and chemokines [48]. Adipose fibrosis is typically associated with increased macrophage infiltration and inflammation in adipose tissue [15, 49]. Chronic macrophage-mediated inflammation in obese adipose tissue has been reported to lead to ECM remodelling and fibrosis [16]. Macrophages serve as the primary immune cells within adipose tissue, and they differentiate from monocytes in the peripheral circulation subsequent to their migration into adipose tissue [50]. The quantity of macrophages in adipose tissue is influenced by several factors, including the pace at which monocytes are attracted to the tissue through chemotaxis, the proliferation and death of macrophages, and the migration of macrophages within the tissue. In the early stage of inflammation in adipose tissue, adipocytes release the proinflammatory factor monocyte chemotactic protein-1 (MCP-1), which acts on CCR2 receptors on the surface of peripheral monocytes to mediate monocyte migration to inflamed adipose tissue, further increasing macrophage infiltration into adipose tissue [51, 52]. The activation of myofibroblasts, tissue injury, and fibrosis are increased in chronic inflammatory conditions due to the influx of infiltrating macrophages [21, 52, 53]. Macrophages present in adipose tissue can be categorised into two distinct types: traditionally activated macrophages (M1) and alternatively activated macrophages (M2) [54]. M1- and M2-type macrophages have roles in fibrosis of adipose tissue [54]. In fibrotic adipose tissue, M1-type macrophages are wrapped around dead adipocytes to form a coronal structure, while M2-type macrophages are concentrated mainly in fibrotic areas of adipose tissue [55, 56]. In obese organisms, macrophages were shown to mediate fibrosis in adipose tissue by regulating fibroblast proliferation/differentiation, and the elimination of macrophages reduced the extent of fibrosis [15]. Furthermore, macrophages can recruit fibroblasts by releasing chemokines and directly activate fibroblasts by secreting TGF-β1 to differentiate fibroblasts into myofibroblasts, which secrete large amounts of collagen and α-SMA (actin alpha, smooth muscle aorta) and promote extracellular matrix production [21]. Therefore, the increase in macrophages in adipose tissue drives adipose tissue fibrosis by activating/recruiting fibroblasts.

Sphingolipids are one of the major species of eukaryotic lipids. Cer, Sph, and S1P are the major bioactive sphingolipids that have received the most attention, and their interconversion is called the “sphingolipid rheostat” [57–59]. Within some mammalian cells, the enzymatic activity of sphingomyelinase (SMase) facilitates the conversion of sphingomyelin (SM) into Cer. Additionally, the enzyme ceramidase (CDase) is responsible for catalysing the conversion of Cer into Sph. Furthermore, the production of S1P can be attributed to the enzymatic activity of SPHK [60]. S1P can be reversibly dephosphorylated by S1P/lipid phosphatase (SPP/LPP) to regenerate Sph, which is then converted back to Cer [61].

Cer is the main hub of sphingolipid metabolism and is an integral part of cell membranes. High levels of Cer and increased macrophage infiltration were detected in the adipose tissue of obese patients, suggesting that Cer may exacerbate chronic inflammation in adipose tissue [62]. Similarly, during tumour-induced malignant progression, tumour-derived proinflammatory factors such as TNF-α increase Cer levels in adipose tissue, and the latter further induces an inflammatory reaction within adipose tissue, increasing the expression of MCP-1 and plasminogen activator inhibitor-1 (PAI-1) [63]. In contrast, treatment with myriocin reduced macrophage accumulation in adipose tissue, reduced the generation of the proinflammatory molecules IL-6, MCP-1, and TNF-α and inhibited the formation of adipose Cer [64]. In addition, Cer can accelerate tissue fibrosis by promoting collagen synthesis, and inhibiting Cer can block fibrosis and inflammatory responses [65, 66]. Moreover, phosphorylated ceramide (ceramide 1-phosphate, C1P) is a potent stimulator of macrophage migration [67]. In vitro studies demonstrated that the addition of C1P to cultured Raw 264.7 macrophages effectively stimulated cell migration [68]. Growth differentiation factor-15 (GDF-15) is a stress cytokine that is expressed in adipose tissue in rats and humans and is secreted by adipocytes. It plays an important role in the regulation of inflammatory responses, growth, and cell differentiation [69]. An increase in circulating GDF15 has been reported to contribute to anorexia and weight loss in a preclinical model of cardiac cachexia [70]. GDF-15 expression can be upregulated by Cer, and GDF-15 promotes remodelling of the extracellular matrix through the activation of Smad proteins [71, 72]. Numerous previous studies have shown that GDF-15 has an important role in myocardial fibrosis, pulmonary fibrosis, hepatic fibrosis, and renal fibrosis [73–76]. Thus, GDF-15 may be a factor in adipose tissue remodelling in cardiac cachexia. In addition, researchers are increasingly realising that many aspects of sphingolipid metabolism are related to oxidative stress in the cell. Some have proposed that increased ceramide levels are mechanistically linked to the onset of oxidative stress [77]. Mitochondria are central to energy metabolism, have key roles in oxidative phosphorylation and reactive oxygen species (ROS) and play an important role in adipose tissue homeostasis and remodelling [78]. Studies have shown that dysregulated mitochondrial metabolism or dysregulated ROS generation leads to pathological remodelling of adipose tissue [79–81]. The relationship between ROS and Cer is bidirectional: an increase in ROS activity stimulates Cer production, while Cer can act directly on mitochondria to increase ROS production [77]. Studies of obesity have shown that an increase in adipose tissue ROS accelerates inflammation and fibrosis in fat [82]. Thus, Cer induces ROS production in adipocyte mitochondria, leading to mitochondrial dysfunction, which may also be an important mechanism by which the sphingolipid metabolic pathway leads to adipose tissue remodelling in cardiac cachexia.

S1P acts on specific receptors (S1PRs) in an autocrine or paracrine manner to mediate cell proliferation, migration, survival, apoptosis, and communication, and is engaged in various physiological functions, such as the inflammatory reaction, autoimmunity, fibrosis, and glycolipid metabolism [83]. S1P can facilitate the chemotaxis of monocytes to adipose tissue because it is favourably linked with the levels of chemokines such as MCP-1 and CCL5 in adipose tissue [84]. Previous studies have confirmed the relevance of the SPHK-S1P-S1PR axis in macrophage differentiation, migration, and viability [85]. S1P acts on S1PR1 to mediate macrophage migration in a concentration-dependent manner [86, 87]. Increased S1P concentrations promote macrophage migration and increase tissue IL-6 expression, while administration of the S1PR receptor antagonist FTY720 prevents macrophage recruitment and decreases IL-6 expression [88]. Moreover, suppressing S1P through the knockdown of SPHK decreased the infiltration of macrophages in the adipose tissue of obese mice. This inhibition also reduced the expression of the proinflammatory molecules TNF-α and IL-6, thereby mitigating inflammation in adipose tissue [89]. It was suggested that reducing S1P could inhibit macrophage migration and reduce inflammation in adipose tissue. The SPHK-S1P-S1PR axis is also involved in fibrosis. In models of lung fibrosis, liver fibrosis and kidney fibrosis, activation of the SPHK-S1P-S1PR axis was shown to induce TGF-β-mediated fibrotic processes, and when inhibited, the SPHK-S1P-S1PR axis reduced fibrotic processes [85]. This effect was associated with macrophages; S1P-S1PR signalling could promote liver fibrosis by affecting bone marrow-derived macrophages [90–92], suggesting that S1P could influence macrophage migration and induce tissue fibrosis. In contrast, reducing S1P by knocking down SPHK2 in renal macrophages could inhibit renal fibrosis by reducing macrophage-mediated inflammatory responses [93]. However, to date, whether the sphingolipid metabolic pathway is involved in adipose tissue fibrosis has not been investigated. The present work offers initial observations about the involvement of the sphingolipid metabolic pathway in the development of fibrosis in adipose tissue.

**Fig. 11:**
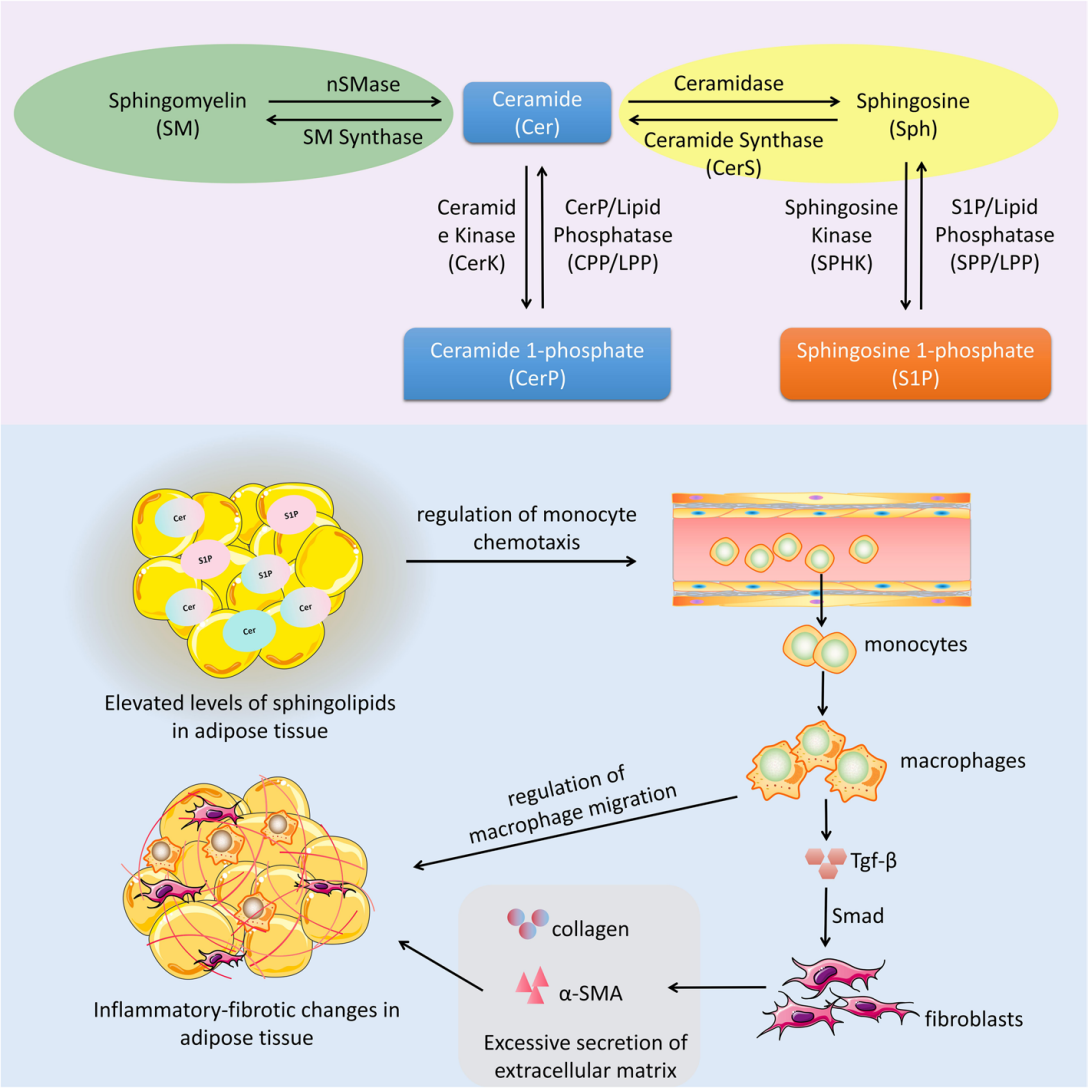
Metabolic processes of Cer and S1P in adipose tissue and mechanisms contributing to adipose tissue remodelling. SMase facilitates the conversion of SM into Cer. CDase is responsible for catalysing the conversion of Cer into Sph. S1P can be attributed to the enzymatic activity of SPHK. S1P can in turn be reversibly dephosphorylated by SPP/LPP to regenerate Sph, which is then converted back to Cer. Ceramide kinase (CerK) catalyzes Cer to produce CerP (C1P), which can in turn be reversibly dephosphorylated by CPP/LPP to regenerate Cer, which is then converted back to SM. Elevated levels of Cer and S1P in adipose tissue lead to the convergence of monocytes from blood to adipose tissue and their differentiation into macrophages. Activation of the Tgf-β/Smad signalling pathway by macrophages causes increased secretion of collagen and α-SMA by fibroblasts, resulting in excessive deposition of extracellular matrix. At the same time, macrophages aggregated to and infiltrated the adipose tissue, causing an inflammatory response in the adipose tissue. Adipose tissue inflammation-fibrosis interactions result in adipose tissue remodelling and dysfunction

These findings suggest that bioactive sphingolipids from adipose tissue can upregulate chemokine expression, promote the migration and accumulation of monocytes/macrophages in adipose tissue, and induce adipose tissue inflammation and fibrosis, resulting in adipose tissue remodelling and dysfunction (Fig. 11).

### Study strengths and limitations

The strength of this study is that combined multiomics studies showed the molecular mechanism of adipose tissue remodelling in cardiac cachexia for the first time, bringing fresh ideas for the clinical development and therapy of new medications for cardiac cachexia. This study also has some limitations.This study did not consider the impact of MCT on adipose tissue metabolism. However, it is worth noting that there is a lack of pertinent literature exploring the influence of MCT on adipose tissue metabolism. Furthermore, this study only tentatively revealed that high levels of sphingolipids in adipose tissue from cardiac cachexia rats were associated with inflammatory-fibrotic changes, but whether the increase of S1P and Cer directly or indirectly caused cardiac cachexia adipose fibrosis still needs further study. In addition, the data in this study pertained only to male rats, and since female rats were not used in the study, the observations were limited.

## Conclusion

In conclusion, through the combined application of transcriptomics, metabolomics, and lipidomics, this study has provided a better knowledge of the molecular mechanisms driving the poor metabolism of cardiac cachexia adipose tissue. The results suggested that the sphingolipid metabolism pathway was the key pathway of adipose tissue remodelling. Adipose tissue remodelling is the pathological basis for adipose tissue dysfunction in cardiac cachexia and is commonly linked with increased macrophage infiltration and increased levels of inflammation in adipose tissue, and abnormal changes in S1P and Cer metabolism may play a crucial role in macrophage differentiation, migration, and inflammatory and fibrotic changes in adipose tissue. From a clinical perspective, this study suggests that inhibiting adipose tissue inflammation and fibrosis may help counteract disruption of energy balance in cardiac cachexia thereby improving patient survival. This study also addressed the treatment bottleneck of cardiac cachexia and identified targets to restore adipose tissue homeostasis, providing valuable insights for the development of novel therapeutic strategies for cardiac cachexia in the future.

### Supplementary Information

**Additional file 1. **(Lipidomics data).

**Additional file 2. **(Metabolomics data).

**Additional file 3. **(Western blot).

**Additional file 4. **Transcriptomics data

## Abbreviations

C1P: Ceramide 1-phosphate
CDase: Ceramidase
Cer: Ceramide
CerK: Ceramide kinase
DEGs: Differentially expressed genes
DELs: Differentially expressed lipids
DEMs: Differentially expressed metabolites
ECM: Extracellular matrix
FPKM: Fragments per kilobase of exon per million reads mapped
GDF-15: Growth differentiation factor-15
GO: Gene Ontology
GSEA: Gene set enrichment analysis
HE: Hematoxylin and eosin reagent
HF: Heart failure
IL-1β: Interleukin-1β
IL-6: Interleukin 6
KEGG: Kyoto Encyclopedia of Genes and Genomes
LC‒MS: Liquid chromatography‒mass spectrometry
MCP-1: Monocyte chemotactic protein-1
MCT: Monocrotaline
OPLS-DA: Orthogonal partial least squares-discriminant analysis
PAI-1: Plasminogen activator inhibitor-1
PCA: Principal component analysis
ROS: Reactive oxygen species
S1P/SPHP: Sphingosine 1-phosphate
S1PR: Sphingosine 1-phosphate receptor
SM: Sphingomyelin
SMase: Sphingomyelinase
Sph: Sphingosine
SPHK: Sphingosine kinase
TGF-β: Transforming growth factor-β
TNF-α: Tumor necrosis factor-alpha
VIP: First principal component
