# Supplementary figures and images for: A comprehensive multiomics approach reveals that high levels of sphingolipids in cardiac cachexia adipose tissue are associated with inflammatory and fibrotic changes
Source: Lipids Health Dis. 2023 Dec 1;22:211. doi: 10.1186/s12944-023-01967-0 (PMC10691093; doi:10.1186/s12944-023-01967-0)

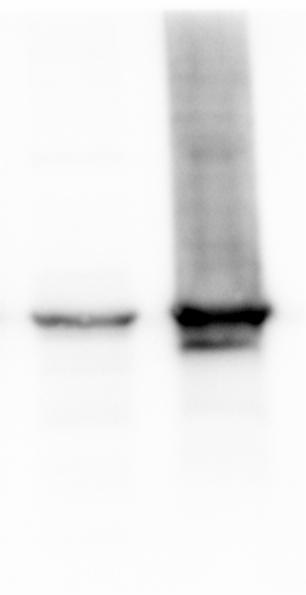

Supplement: Supplementary file 3 — Additional file 3. (Western blot). [file 12944_2023_1967_MOESM3_ESM.zip › western blotting/S1PR1/S1PR1(1).jpg]

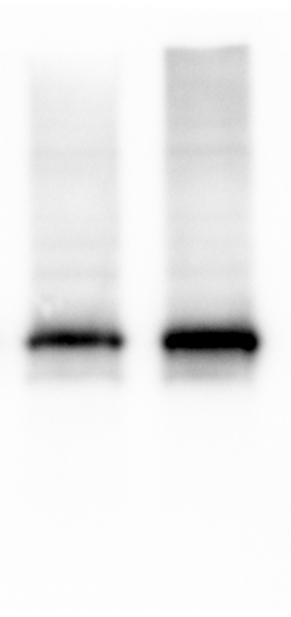

Supplement: Supplementary file 3 — Additional file 3. (Western blot). [file 12944_2023_1967_MOESM3_ESM.zip › western blotting/S1PR1/S1PR1(2).jpg]

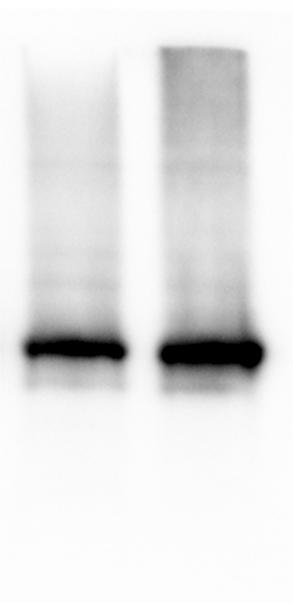

Supplement: Supplementary file 3 — Additional file 3. (Western blot). [file 12944_2023_1967_MOESM3_ESM.zip › western blotting/S1PR1/S1PR1(3).jpg]

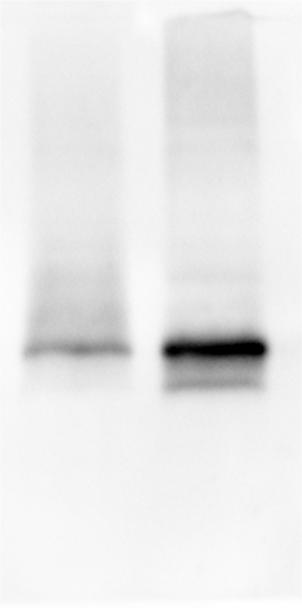

Supplement: Supplementary file 3 — Additional file 3. (Western blot). [file 12944_2023_1967_MOESM3_ESM.zip › western blotting/S1PR2/S1PR2(1).jpg]

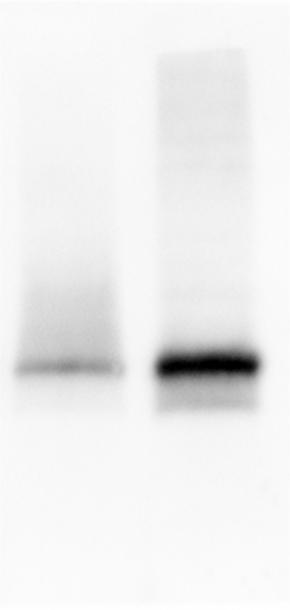

Supplement: Supplementary file 3 — Additional file 3. (Western blot). [file 12944_2023_1967_MOESM3_ESM.zip › western blotting/S1PR2/S1PR2(2).jpg]

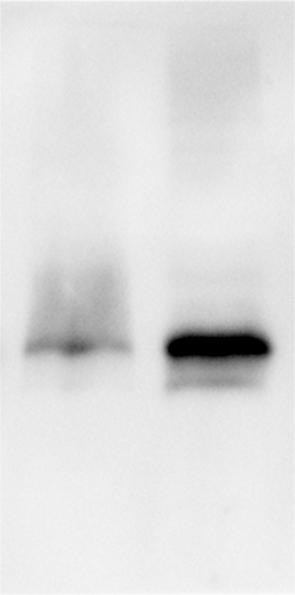

Supplement: Supplementary file 3 — Additional file 3. (Western blot). [file 12944_2023_1967_MOESM3_ESM.zip › western blotting/S1PR2/S1PR2(3).jpg]

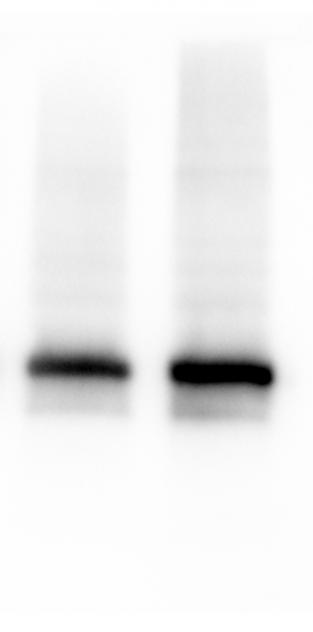

Supplement: Supplementary file 3 — Additional file 3. (Western blot). [file 12944_2023_1967_MOESM3_ESM.zip › western blotting/SPHK/SPHK(1).jpg]

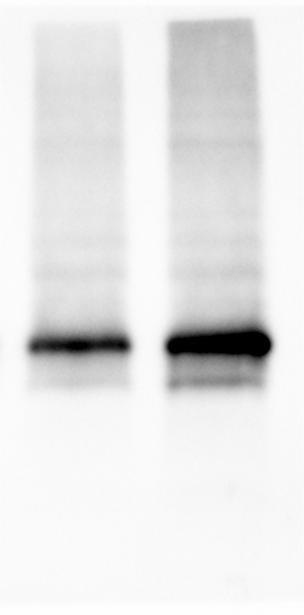

Supplement: Supplementary file 3 — Additional file 3. (Western blot). [file 12944_2023_1967_MOESM3_ESM.zip › western blotting/SPHK/SPHK(2).jpg]

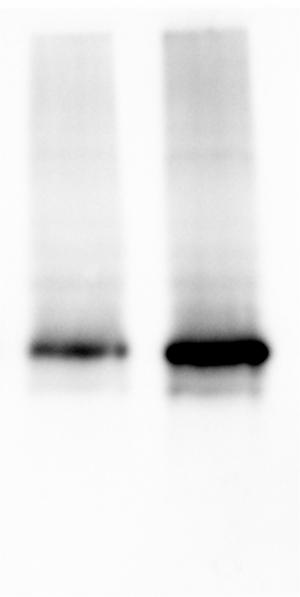

Supplement: Supplementary file 3 — Additional file 3. (Western blot). [file 12944_2023_1967_MOESM3_ESM.zip › western blotting/SPHK/SPHK(3).jpg]

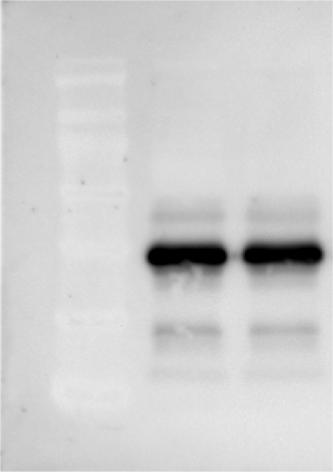

Supplement: Supplementary file 3 — Additional file 3. (Western blot). [file 12944_2023_1967_MOESM3_ESM.zip › western blotting/β-actin/β-actin(1).jpg]

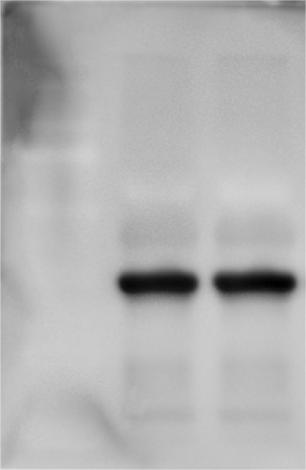

Supplement: Supplementary file 3 — Additional file 3. (Western blot). [file 12944_2023_1967_MOESM3_ESM.zip › western blotting/β-actin/β-actin(2).jpg]

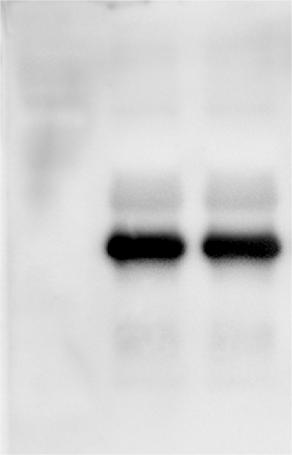

Supplement: Supplementary file 3 — Additional file 3. (Western blot). [file 12944_2023_1967_MOESM3_ESM.zip › western blotting/β-actin/β-actin(3).jpg]
